# Diabetic macrophage small extracellular vesicles-associated miR-503/IGF1R axis regulates endothelial cell function and affects wound healing

**DOI:** 10.3389/fimmu.2023.1104890

**Published:** 2023-05-23

**Authors:** Jianqiang Wang, Yuanshan Han, Fang Huang, Liuhuan Tang, Jianfei Mu, Ying Liang

**Affiliations:** ^1^Molecular Nutrition Branch, National Engineering Research Center of Rice and By-Product Deep Processing/College of Food Science and Engineering, Central South University of Forestry and Technology, Changsha, Hunan, China; ^2^Scientific Research Department, The First Hospital of Hunan University of Chinese Medicine, Changsha, Hunan, China

**Keywords:** diabetic foot ulcer (DFU), endothelial cell (EC), miR-503, IGF1R, macrophage M1 polarization, small extracellular vesicles (sEVs)

## Abstract

Diabetic foot ulcer (DFU) is a break in the skin of the foot caused by diabetes. It is one of the most serious and debilitating complications of diabetes. The previous study suggested that dominant M1 polarization during DFU could be the leading reason behind impaired wound healing. This study concluded that macrophage M1 polarization predominates in DFU skin tissue. iNOS was increased in HG-induced M1-polarized macrophages; conversely, Arg-1 was decreased. Macrophage pellets after HG stimulation can impair endothelial cell (EC) function by inhibiting cell viability, tube formation and cell migration, indicating M1 macrophage-derived small extracellular vesicles (sEVs) -mediated HUVEC dysfunction. sEVs miR-503 was significantly upregulated in response to HG stimulation, but inhibition of miR-503 in HG-stimulated macrophages attenuated M1 macrophage-induced HUVEC dysfunction. ACO1 interacted with miR-503 and mediated the miR-503 package into sEVs. Under HG stimulation, sEVs miR-503 taken in by HUVECs targeted IGF1R in HUVECs and inhibited IGF1R expression. In HUVECs, miR-503 inhibition improved HG-caused HUVEC dysfunction, whereas IGF1R knockdown aggravated HUVEC dysfunction; IGF1R knockdown partially attenuated miR-503 inhibition effects on HUVECs. In the skin wound model in control or STZ-induced diabetic mice, miR-503-inhibited sEVs improved, whereas IGF1R knockdown further hindered wound healing. Therefore, it can be inferred from the results that the M1 macrophage-derived sEVs miR-503 targets IGF1R in HUVECs, inhibits IGF1R expression, leads to HUVEC dysfunction, and impedes wound healing in diabetic patients, while packaging miR-503 as an M1 macrophage-derived sEVs may be mediated by ACO1.

## 1 Introduction

Diabetic foot is a common and debilitating complication of diabetes, characterized by an ulcerated foot in diabetic patients that is associated with neuropathy and/or peripheral vascular disease of the lower extremities (1, 2). The term “diabetic foot ulcer” (DFU) refers to a break in the skin on the foot that does not heal rapidly due to diabetes. Several factors lead to the break in the skin, and once the ulcer has formed, multiple factors prevent its normal healing (3). The global incidence of diabetes mellitus and the increased life expectancy of people with diabetes have led to the growing prevalence of DFU; however, the lack of efficient treatment has heaped drastic financial burdens on patients, their families, and society (4).

Macrophages are key players in wound healing and have a continuous function at the wound site starting at the time of injury (5, 6). The macrophage population is heterogeneous, which is related to the different responses of macrophages to microenvironmental signals. In the presence of harmful stimuli, the resident macrophages and monocyte-derived macrophages of the tissues involved in the inflammatory process are polarized into 2 subgroups, M1 (pro-inflammatory) and M2 (anti-inflammatory) phenotypes (5, 7, 8), known as macrophage polarization. M1 macrophages are prevalent during the early phases of inflammation. It causes bacterial phagocytosis and the secretion of proinflammatory cytokines, including TNF-α and IL-6. In diabetic wounds, the switch from M1- to M2-like phenotypes are impaired, and the expression of M2 marker genes (including Arg1) is dramatically decreased (5). The M1 phenotype is characterized by a high production of proinflammatory cytokines, including inducible nitric oxide synthase (iNOS), IL-6, IL-12, and TNF-α, upon IFN-γ and LPS (9). The M2 phenotype produces large amounts of anti-inflammatory mediators; enhances angiogenesis mediators, including arginase-1 (ARG1), IL-10, TGF-β1, and VEGF; and exerts an essential effect on tissue repair, reconstruction and tumors (10, 11). Excessive inflammatory reaction, accompanied by M1 macrophage prolonged accumulation and increased proinflammatory cytokines, could lead to the delayed healing of diabetic wounds. Therefore, dominant M1 polarization during DFU could be the main factor behind wound healing.

Macrophage dysfunction can also result in the communication failure between macrophages and epithelial, endothelial, fibroblast, stem or tissue progenitor cells, leading to a state of persistent damage (12, 13). Diabetes can exert effects on wound healing through multiple pathophysiological mechanisms, including persistent inflammation and hypoxia, cellular dysfunction, impairment of angiogenesis/neovascularization, and neuropathy (14). Vascular endothelial cells (ECs) proliferate and chemoattract as they are stimulated by numerous angiogenic factors. They also extend and migrate to the repair area, leading to the granulation of the wound surface and promoting the repair of the capillary network, thereby exerting a critical effect on the wound-healing process (15, 16). Given the critical roles of these key events in the wound-healing process during DFU, it is speculated that M1 macrophages may affect the function of vascular endothelial cells, thereby hindering wound healing.

MicroRNAs (miRNAs) belong to a class of non-coding RNAs which are ~22 nucleotides in length. MiRNAs predominately act as post-transcriptional gene regulators. While each miRNA is predicted to have multiple potential target messenger RNAs (mRNAs), a single gene can also be modulated by several miRNAs, thereby increasing the complexity through which miRNAs can fine-tune gene expression (17). Several miRNAs have been reported to exert a role in diabetic wound repair. MiR-132 expression is reduced in DFU compared to normal skin wounds. MiR-132 played an anti-inflammatory role by blocking inflammatory cytokines secretion (18). miR-21 promoted the M1 macrophage polarization in the diabetic wound and was highly expressed in In DFUs skin tissues (19, 20). The sEVs (also refer to as exosomes by some researchers) are a class of extracellular vesicles that are 30 to 150 nm in diameter and highly enriched in tetraspanins (21). Tetraspanins, including CD9, CD63 and CD81 are especially enriched in the membrane of sEVs and are often used as sEVs biomarkers (22). sEVs result from the fusion of multivesicular bodies and are involved in cell-to-cell communication through the transmission of intracellular cargos, such as proteins, mRNAs, miRNAs, and long non-coding RNAs (lncRNAs) (23, 24). The sEVs mediated transfer of miRNAs plays a well-documented role in macrophage functions and related inflammatory diseases and cancers. For example, macrophages regulate the invasive nature of breast cancer cells through microvesicles -mediated delivery of oncogenic miRNAs (25). Serum sEVs-derived miR-155 enhanced macrophage proliferation activity and inflammation by binding to SHIP1 and SOCS1, respectively in cases of acute lung inflammation (26). Nevertheless, the function and mechanism of macrophage-derived sEVs and macrophage sEVs-miRNA in DFU wound healing remain unclear.

In this study, macrophage polarization status in DFU was first investigated in clinical tissue samples and high-glucose (HG)-stimulated macrophages by assessing the levels of M1 polarization marker (iNOS) and M2 polarization markers (CD163 and Arg-1). The sEVs from M1-polarized macrophages were extracted and were examined for the effects of M1 macrophage-derived sEVs upon HUVEC viability, tube formation, migration, angiogenesis and cell adhesion-related factors. The sEVs associated miRNAs that potentially mediate macrophage-EC communication were analyzed, and the effects of sEVs miRNA on HUVEC functions were investigated. Regarding the underlying molecular mechanism, the factor that might mediate the miRNA packaging into sEVs was analyzed, and the analyzed interaction was validated. Moreover, the miRNA downstream target in HUVECs was analyzed. The predicted miRNA binding to the target was validated. The dynamic effects of the sEVs miRNA/EC mRNA axis on HG-stimulated HUVEC functions and skin wound healing model in diabetic mice were subsequently investigated.

## 2 Materials and methods

### 2.1 Clinical sampling

The skin samples around the diabetic lesion were harvested from 12 diabetic patients who had undergone amputation surgery. Healthy control samples were harvested from 12 non-diabetic patients who underwent reconstructive surgery having sustained foot injuries. The samples were quickly cut into small strips, fixed in 4% paraformaldehyde overnight, and embedded in paraffin. Each paraffin block was serially cut into 4-μm slices and used for subsequent investigations. The sampling procedure was carried out with the approval of the Ethics Committee of The First Hospital of the Hunan University of Chinese Medicine. Informed consent was signed by each patient enrolled.

### 2.2 Hematoxylin and eosin and masson staining

Skin sample sections were deparaffinized and hydrated, followed by staining with H&E and Masson staining as stipulated by the manufacturer. For H&E staining, sections were incubated with hematoxylin solution for 10 min, hydrochloric acid ethanol differentiation for 15s, running tap water for 10 min and eosin solution for 1 min. For Masson staining, the sections were incubated with Weigert’s iron hematoxylin working solution for 10 min, Biebrich scarlet-acid fuchsin solution for 10 min, running tap water for 10 min, phosphomolybdic-phosphotungstic acid solution differentiation for 10 min, aniline blue solution for 5 min and acetic acid solution for 2 min. The sections were finally observed under a light microscope at 200x magnification (Nikon, Japan).

### 2.3 Real-time quantitative reverse transcription PCR

A Trizol Reagent (Ambion, Austin, TX, USA) was used to extract total RNA from tissue samples or cells as directed by the manufacturer. Briefly, the extracted RNA samples were assessed for RNA concentration and quality using Nano Drop Lite Spectrophotometer 120 V (Thermo Fisher Scientific, Waltham, MA, USA). The Primescript RT reagent Kit (Takara, Kyoto, Japan) was subsequently applied to reversely transcribe the total RNA into cDNA, and the SYBR® premix Ex Taq TM kit (Takara) was employed to conduct qRT-PCR. β-actin or U6 acted as the internal control. The relative expression of target factors was analyzed using the 2^−ΔΔCt^ method. The primer sequence is listed in **Table S1**.

### 2.4 Immunoblotting

Cells or sEVs were collected at the indicated time-points and lysed in SDS lysis buffer (Beyotime, Shanghai, China), followed by centrifugation at 14,000×g for 10 min at 4 °C. Nuclear and Cytoplasmic Protein Extraction Kit (Beyotime) was used to separate nuclear or cytoplasmic proteins. Following separation on 10% or 15% polyacrylamide gels, 30 μg proteins were transferred to PVDF membranes (Merck Millipore, Burlington, MA, USA). The blots were blocked at ambient temperature for 1 h with 5% BSA, followed by incubation overnight at 4 °C with primary antibodies against iNOS, Arg-1, Ang1, Flk1, Vash1, TSP1I, ICAM-1, VCAM-1, CD9, CD63, CD81, Calnexin, ACO1, and IGF1R. The protein β-actin (total protein), Histone H3 (nucleoprotein) and Alix (sEVs) were used as endogenous control. All the primary antibodies were procured from Abcam, USA. The membranes were then probed with horseradish peroxidase (HRP)-labeled secondary antibodies (HPR-labeled goat-anti rabbit or mouse IgG. Beyotime, Shanghai, China) for 1 hour at ambient temperature. The enhanced chemiluminescence (ECL) method was ultimately used for development.

### 2.5 Cell lineages and cell culture

Human THP-1 cell line (TIB-202) was obtained from ATCC and cultivated in the Roswell Park Memorial Institute (RPMI) 1640 media (Thermo Fisher Scientific) containing 5.5 mM D-glucose, 1 mM sodium pyruvate and 10% heat-inactivated fetal bovine serum (FBS) (Thermo Fisher Scientific) and 1% kanamycin. Human umbilical vascular endothelial cell lines (HUVEC) were procured from ATCC (CRL-1730™) and cultivated in F-12K Medium (ATCC) containing 10% FBS, 0.1 mg/mL heparin, and 30 µg/mL ECGS. Mouse macrophage 264.7 and endothelial cell line C166 were obtained from ATCC and cultivated in DMEM media with 10% FBS. Cells were cultured at 37°C in a 5% CO_2_ atmosphere.

### 2.6 Cell differentiation and macrophage polarization

THP-1 cells were cultured for 48 h with 10 ng/mL PMA and after one day the media was replaced with a fresh RPMI 1640 media (with 5.5 mM D-glucose) for monocyte THP-1 differentiation into M0 macrophage. For M0 macrophage polarization into the M1 subtype, monocyte-derived M0 macrophages were exposed to high levels of glucose. As a control, M0 macrophages were cultured with a standard culture medium. High-glucose (HG)-stimulated M0 macrophages were incubated in RPMI-1640 medium supplemented with 30 mM D-glucose for 3 days (27, 28). M0 macrophages were collected with sterile cell scrapers on day 4, and the cell culture supernatant was harvested from each well for subsequent analyses. For mouse macrophage polarization, the RAW264.7 cells (considered as M0 mouse macrophages) were exposed to RPMI-1640 with 30 mM D-glucose for 3 days.

### 2.7 Immunofluorescent staining

Cells grown on coverslips or tissue sections were fixed with 4% paraformaldehyde, followed by permeabilization with 0.1% Triton X-100, blocking with 3% BSA, and incubation overnight at 4 °C with anti-iNOS (dilution 1:500, ab178945, Abcam, Cambridge, MA, USA), anti-CD68 (dilution 1:100, ab213363, Abcam), or anti-Arg-1 (dilution 1:200, ab96183, Abcam). The samples were subsequently incubated at ambient temperature for 30 min in light-deprived conditions with an Alexa Fluor 488-labeled anti-rabbit IgG (dilution 1:500, A-11008, Invitrogen, Waltham, MA, USA) followed by treatment with DAPI (Invitrogen) for nucleus staining. Staining results were photographed and analyzed under an inflorescence microscope (Olympus, Japan).

### 2.8 Enzyme-linked immunosorbent assay

The culture medium was collected from different groups and ELISA kits were employed as directed by the manufacturer to determine the levels of IL-6, MCP-1, IL-4, and IL-10 in the medium. Human IL-6, MCP-1, IL-4, and IL-10 ELISA Kits were procured from Abcam.

### 2.9 sEVs extraction

After culturing the macrophages at 37°C in a humidified atmosphere containing 5% CO_2_ for 72 h, ExoQuick-TC (System Biosciences, Palo Alto, CA, USA) was applied to extract the sEVs following the aforementioned methods (29). The cell culture suspension was centrifugated at 3000 g for 15 min. Then, the supernatant was transferred to a 10 mL sterile centrifuge tube, and the ExoQuick-TC reagent was added to the supernatant. The mixtures were subsequently refrigerated overnight (at least 12 h). The supernatant was centrifugated at 1500 g for 30 min and aspirated. A 1500 g centrifugation was performed for 5 min to remove the remainder of the ExoQuick-TC solution. The sEVs pellet was ultimately re-suspended and kept pending further experiments. 2 μg of sEVs (equivalent to those obtained from ~5×10^6^ M0 or HG treated M0 cells) based on protein concentrations measured with a Pierce™ BCA protein assay kit (Thermo Fisher Scientific) was treated with 2×10^5^ HUVECs for 24 h.

### 2.10 Uptake of sEVs by HUVECs

Macrophage-derived sEVs were applied with a PKH67 dye (Sigma-Aldrich, MA, USA) for labeling following the aforementioned method (30). Briefly, 20 μl of sEVs were diluted in 1 ml of diluent C and 6 μl of PKH67 dye. The labeled sEVs were washed in PBS for 70 min at 100,000 g., HUVECs were plated in a 6-well plate (1×10^4^ cells/well) and cultured for 24 h to determine the uptake of sEVs into HUVECs. The adherent HUVECs were rinsed thrice by PBS, and each media supplemented with PKH67-labeled sEVs, non-labeled sEVs, or PKH67 dye without the sEVs sample was added into each well. The cells were cultivated at 37°C in a humidified atmosphere containing 5% CO_2_ for 24 h, rinsed thrice with PBS, and added with 4% paraformaldehyde solution for fixation at ambient temperature for 10 min. DAPI (Life Technologies) was employed for nucleus staining. Finally, after washing the slides with PBS thrice, the cells were observed under an inflorescence microscope (Olympus, Japan) after being washed thrice with PBS.

### 2.11 HUVECs treatments

M0 macrophage, HG stimulated M0 macrophage, 10 μM GW4869 (sEVs generation inhibitor, Sigma) pretreated HG stimulated M0 macrophage or HG stimulated transfected M0 macrophage were cultured for 72 h, the condition media (CM) was retrieved for sEVs extraction or HUVECs direct incubation. HUVECs were subsequently incubated with different sources of sEVs or CM (the collected M0-CM were added additional D-glucose to keep the glucose concentration consistent with HG+M0-CM) for 24 h. HUVECs were then harvested for further experiments, including cell viability, tube formation, migration, RT-PCR and immunoblotting analysis.

### 2.12 HUVECs and macrophage transfection

miR-503 mimics/inhibitor was synthesized and retrieved from GenePharma, Shanghai China. The mimics negative control (NC) or inhibitor NC was used as a negative control. The final concentration of 50 nM miRNA mimics/inhibitor was transfected in HUVECs and macrophages using Lipofectamine 3000 Reagent (Thermo Fisher Scientific, Waltham, MA, USA) as directed by the manufacturer. Briefly, cells were seeded in a 6-well plate and after reaching 70% confluency, the cells were incubated with a mix of RNA or DNA-lipo3000 complex (250 μl per well). 48 h after transfection, the cells or CM were collected for further experiments. The final concentration of 2 μg/mL of sh-IGF1R vector (GenePharma) was transfected into HUVECs as mentioned above to achieve IGF1R knockdown in HUVECs. The vector containing scramble sh-RNA sequence was used as a negative control (sh-NC). The sequence is listed in **Table S2**.

### 2.13 Cell counting kit-8 assay

Different treated HUVECs were planted in a 96-well plate at a density of 8 × 10^3^ cells per well, followed by incubation overnight at 37°C. After treatment with sEVs under different conditions for 24 h, CCK-8 solution (Beyotime, Shanghai, China) was added to each well to incubate the cells for 2 hours. The absorbance value was measured at a wavelength of 490 nm at the end of the incubation.

### 2.14 Tube formation

Matrigel Basement Membrane Matrix (BD Biosciences, Franklin Lakes, NJ, USA) was diluted with EBM-2 medium and coated in 24-well plates at 37°C for 1 h. 5 × 10^4^ different treated HUVECs were subsequently planted into the F-12K media upon Matrigel. The angiogenesis capability of HUVECs was assessed after 6 h. The number of tubes and nodes in the tubular structures was measured after incubation.

### 2.15 Wound healing

Different treated HUVECs were inoculated into a six-well cell culture plate and culture to ~100% confluence for wound healing test. After starving for 6 h, the cell monolayer was interrupted by introducing a scratch using the tip of a sterile 200 μL pipette to create an artificial uniform wound. After 24 h, the wound was observed under a microscope and the migrating area was analyzed.

### 2.16 RNA pull-down and RNA binding protein immunoprecipitation assays

The Biotin-coupled miRNA pull-down assay was carried out as previously described (31). Briefly, after M1 polarization by HG stimulation, the sEVs were extracted from the culture medium and sEVs protein isolation by SDS lysis buffer. A Nuclear and Cytoplasmic Protein Extraction Kit (Beyotime) was used to isolate the nucleus and cytosol proteins. The isolated proteins were subsequently incubated with 100 pmol 3’ end biotinylated miR-503, mutant miRNA-503 or control RNA (poly G) (RiboBio, Guangzhou, China) with rotation at 4°C overnight. The biotin-coupled RNA protein complex was incubated at 4°C for 4 h with streptavidin-coated magnetic beads (Life Technologies) under rotation. The precipitates were rinsed 5 times and heated with the SDS buffer. The abundance of ACO1 protein in the pull-down precipitates was evaluated by immunoblotting.

The Magna RIP RNA-Binding Protein Immunoprecipitation Kit (Millipore, Bedford, MA) was used to perform RIP experiments. Briefly, a compacted pellet (about 1×10^7^ cells) was resuspended in an equal volume of RIP Lysis Buffer (approximately 100 μL) supplemented with protease and RNase inhibitors. 20 μL of lysate was stored as the input sample. 100 μL of cell lysates were incubated at 4°C overnight with 5 μg of control mouse IgG or antibody against ACO1 (WH0000048M1, Sigma-Aldrich) under rotation. Next, 30 μL of A/G protein magnetic beads was added, followed by incubation at 4°C for 4 hours. Following treatment with proteinase K, miRNeasy Micro Kit (Qiagen) was applied to extract the immunoprecipitated RNAs and reverse transcription was operated with Prime- Script RT Master Mix (TaKaRa). qRT-PCR assay was conducted to detect the level of miR-503.

### 2.17 Mouse skin wound model and treatments

All the animal experiments were carried out with the approval of The First Hospital of the Hunan University of Chinese Medicine. A total of 48 female Balb/c mice (2-month-old, weight 25-30g) were selected in this study. Mice were given an injection of 50 mg/kg intraperitoneal streptozotocin (STZ; Sigma) to elicit a diabetes model in mice. STZ was freshly dissolved in 0.1 M phosphoric acid-citrate buffer (pH 4.5). Diabetic mice were fasted for 18 h and then intraperitoneally injected with STZ daily for five consecutive days, while normal control mice (non-DM control; n=8) were injected with the same amount of phosphoric acid-citrate buffer. The blood glucose level was measured 3 days later, and mice with blood glucose concentrations higher than 16.67 mM (n> 30) were diagnosed with diabetes. Diabetic mice were monitored for 2 weeks prior to skin wounding.

Sodium pentobarbital (50 mg/kg; Sigma) was intraperitoneally injected into diabetic mice or control mice for anesthesia. Following general anesthesia and shaving of the dorsal hair, two 8-mm full-thickness excisional wounds were made on the dorsum of each mouse. Mice in the experimental group were subsequently randomly separated into 5 treatment groups: DM+sEVs group (wound treated with no transfected HG-stimulated macrophage’ sEVs), inhibitor NC sEVs + sh-NC group (wounds treated with inhibitor NC transfected HG-stimulated M0 macrophage’ sEVs (50 μg) plus sh-NC lentivirus (5×10^7^ TU) in 100 μL PBS), miR-503 inhibitor sEVs + sh-NC group (wounds treated with miR-503 inhibitor transfected HG-stimulated M0 macrophage’ sEVs plus sh-NC lentivirus in 100 μL PBS), inhibitor NC sEVs + sh-IGF1R group (wounds treated with inhibitor NC transfected HG-stimulated M0 macrophage’ sEVs plus sh-IGF1R lentivirus in 100 μL PBS), miR-503 inhibitor sEVs + sh-IGF1R group (wounds treated with miR-503 inhibitor transfected HG-stimulated M0 macrophage’ sEVs plus sh-IGF1R lentivirus in 100 μL PBS). The mouse M0 macrophage (264.7 cells) culture, HG stimulation, transfection and sEVs isolation were performed as described in sections 2.6, 2.9, and 2.12. The lentiviruses were purchased from Genechem, Shanghai, China.

Mice were injected with the above-mentioned reagent (25 μL per site) subcutaneously at 4 injection sites at the four mid-point of the wound edge. After the operation, the defects were covered by a skin patch (3 M) and all the mice were sent back to the biosafety facility and were given free access to food and water. Photographs of the wound were taken at 0, 3, 6, 9, and 12 days following the injury, and the wound size was measured using a caliper. The calculation formula for wound size reduction is wound size reduction (%) = (A0-At)/A0×100; in the formula, A0 denotes the initial wound area and At is the wound area at the specified time point. The mice were sacrificed 12 days following surgery, and skin specimens were harvested. The undersurface of the epidermis was observed, and pictures were taken to observe the new blood vessels. Skin tissues were collected for qRT-PCR and Immunoblotting analyses.

### 2.18 Statistical analysis

Data analyses and graphing were conducted using GraphPad PRISM software (GraphPad Software, Inc.). Animal group sizes were chosen based on previous experience with the animal models used. No data points were excluded from the analysis in any experiment. Data were analyzed using SPSS17.0 (IBM, Armonk, NY, USA). Shapiro–Wilks test was used to explore whether data are normally distributed. Brown-Forsythe test was used for group variances analysis. The t-test was used for comparison between the two groups. The Kruskal–Wallis test was used for non-parametric statistical analysis. For comparing several groups with unequal variances, the data were analyzed using one-way ANOVA and Dunnett’s T3 test. If the data belongs to equal variances, one-way ANOVA followed by an LSD test was used. Results were expressed as mean ± s.e.m. p<0.05 was considered a statistically significant difference.

### 2.19 Ethical approval and consent to participate

All animal experiment protocols, and the sampling procedure was carried out with the approval of the Ethic Committee of The First Hospital of the Hunan University of Chinese Medicine. All study participants provided written informed consent prior to participation in the study.

## 3 Results

### 3.1 HG-induced macrophage M1 polarization impairs HUVEC functions

**Figure 1:**
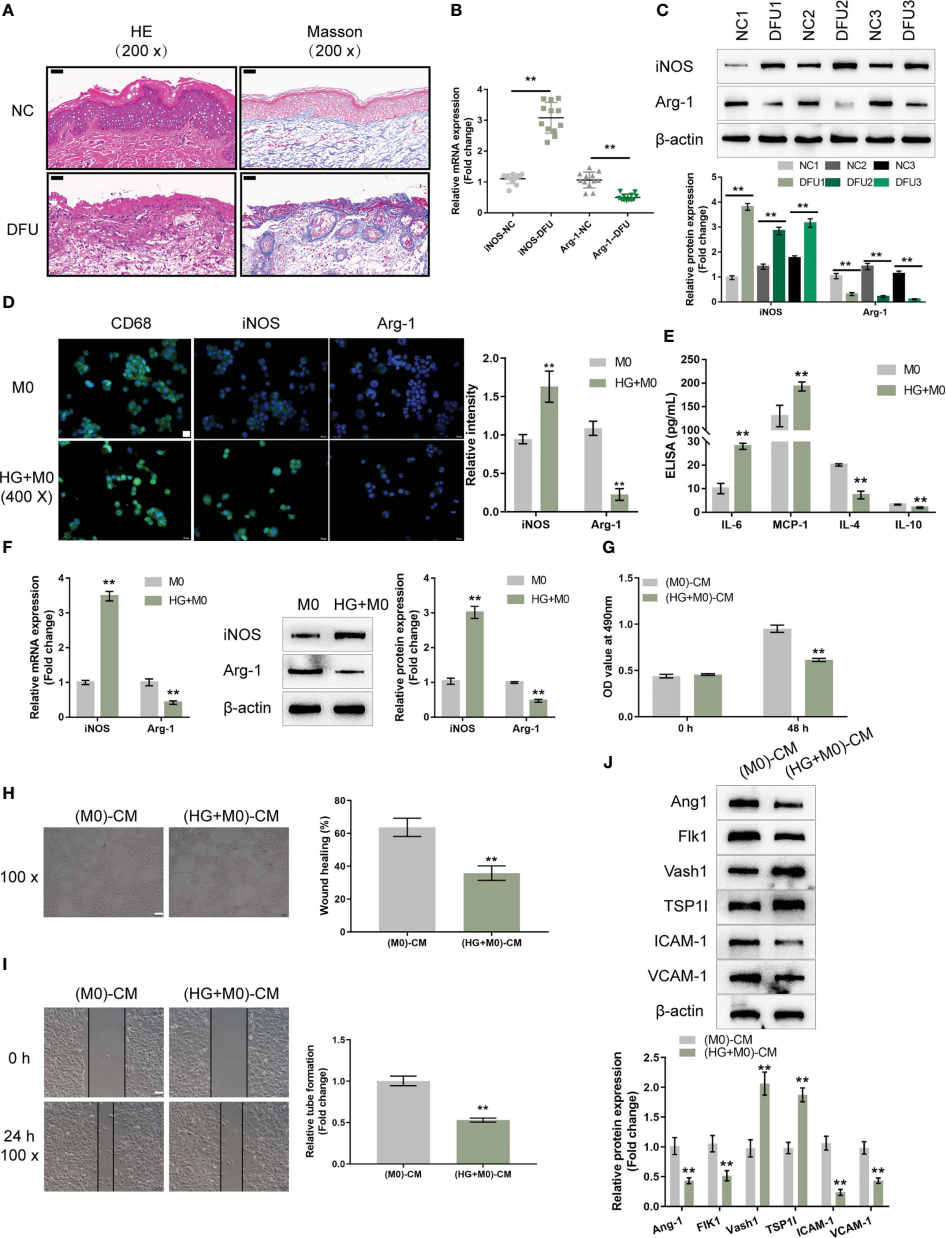
High-glucose-induced macrophage M1 polarization impairs endothelial cell functions **(A)** The histopathological characteristics of normal control (NC; n=12) and diabetic foot ulcer (DFU; n=12) clinical samples were examined using H&E and Masson staining. **(B)** The mRNA expression of iNOS and CD163 in NC and DFU samples was examined using qRT-PCR. **(C)** The protein levels of iNOS and CD163 in NC and DFU samples were examined using Immunoblotting. THP-1 cells were treated with PMA at 5 ng/mL for 48 h to induce M0 differentiation, treated with 30 mM glucose (high-glucose, HG) to induce M1 polarization, and examined for the levels of CD68, iNOS, and Arg-1 using Immunofluorescent staining (IF staining) **(D)**; the levels of IL-6, MCP-1, IL-4, and IL-10 in the culture medium using ELISA **(E)**; the mRNA expression and protein levels of iNOS and Arg-1 using qRT-PCR and Immunoblotting, respectively **(F)**. THP-1 cells were treated with PMA at 5 ng/mL for 48 h to induce M0 differentiation, treated with 30 mM glucose (high-glucose, HG) for 72 h to induce M1 polarization, and collected for the conditioned culture medium (M0)-CM or (HG+M0)-CM) to incubate HUVECs. The cell viability of HUVECs was examined using CCK-8 assay **(G)**; tube formation capacity of HUVECs was determined using tube formation assay **(H)**; HUVECs cell migration was determined using Wound healing assay **(I)**; the protein levels of Ang1, Flk1, Vash1, TSP1I, ICAM-1, and VCAM-1 were determined using Immunoblotting assay **(J)**. n=3, ** p<0.01.

To investigate the polarization status of macrophages in DFU, firstly, H&E and Masson’s staining were performed to examine the histopathological characteristics of normal control and DFU clinical samples. The number of blood vessels in the dermal papilla layer of the skin at the edge of the ulcer wound in DFU samples was significantly reduced. The number of fibroblasts was reduced and disordered; the collagen was scarce and disordered. Some of the collagens were deformed and broken, and infiltration of inflammatory cells was observed (**Figure 1A**). As shown by qRT-PCR, iNOS (M1 marker) mRNA expression was increased within DFU samples, whereas CD163 (M2 marker) was downregulated compared with the normal samples (**Figure 1B**). Consistently, the protein levels of iNOS were increased in DFU samples, whereas CD163 levels were decreased (**Figure 1C**). Thus, macrophage M1 polarization is frequently observed in DFU samples.

To investigate the macrophage polarization in DFU, THP-1 cells were stimulated with PMA at 5 ng/mL for 48 h to induce M0 differentiation. These cells were treated with 30 mM glucose (high-glucose, HG) to induce M1 polarization, and examined for the levels of CD68 (macrophage marker), iNOS (M1 marker), and Arg-1 (M2 marker). As demonstrated by IF staining, HG significantly induced the CD68 and iNOS levels but caused no changes in Arg-1 level compared with the PMA-induced M0 group (**Figure 1D**). Consistently, the mRNA expression and protein levels of iNOS were increased in the HG group, whereas Arg-1 levels were decreased, compared with the PMA-induced M0 group (**Figure 1F**). In the culture medium, HG increased the levels of IL-6, MCP-1, and decreased IL-4 and IL-10 (**Figure 1E**). Thus, it is concluded that HG could successfully induce macrophage M1 polarization.

The effects of macrophage polarization on HUVEC functions were subsequently examined. THP-1 cells were stimulated with PMA at 5 ng/mL for 48 h to induce M0 differentiation, these cells were treated with 30 mM glucose (high-glucose, HG) to induce M1 polarization, and the conditioned culture medium was then collected for (M0)-CM or (HG+M0)-CM) to incubate HUVECs for 24 h. HUVECs were subsequently collected for cell viability, tube formation, migration and western blot assays. (HG+M0)-CM significantly inhibited HUVEC viability (**Figure 1G**), tube formation ability (**Figure 1H**), and migratory ability (**Figure 1I**), compared with the M0-CM group. Consistently, (HG+M0)-CM decreased the protein levels of proangiogenic Ang1 and Flk1, increased anti-angiogenic Vash1 and TSP1I, and decreased intercellular adhesion molecule ICAM-1 and vascular cell adhesion molecule VCAM-1, compared with M0-CM group (**Figure 1J**). Thus, HG-induced macrophage M1 polarization impairs HUVEC functions.

### 3.2 sEVs mediate macrophage M1 polarization effects on HUVEC functions

**Figure 2:**
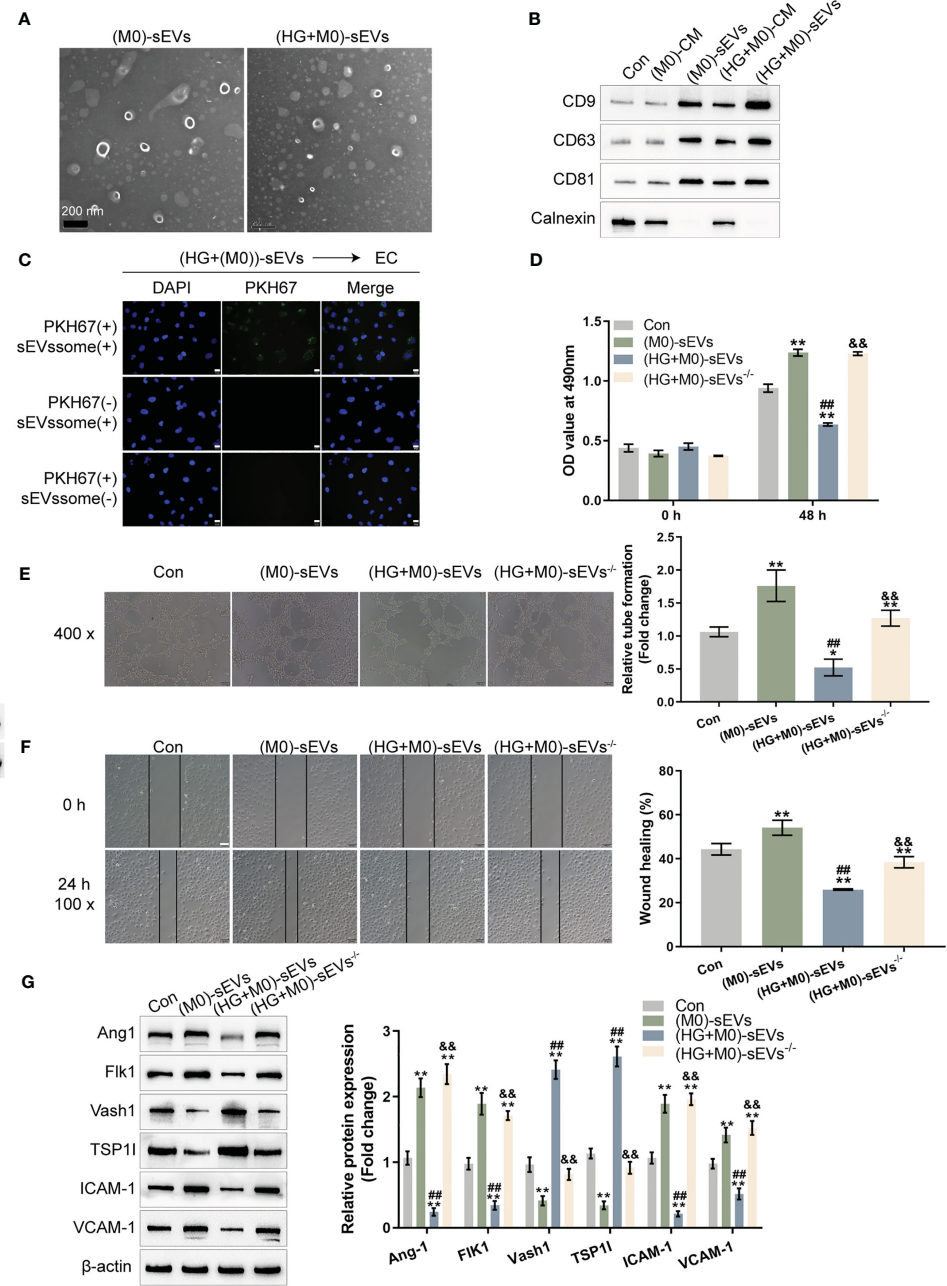
sEVs mediate macrophage M1 polarization effects on HUVEC functions **(A)** THP-1 cells were treated with PMA at 5 ng/mL for 48 h to induce M0 differentiation, treated with 30 mM glucose (high-glucose, HG) to induce M1 polarization, and extracted forsEVs. The sEVs was observed using a transmission electron microscope (TEM). **(B)** Protein samples were extracted from the following five groups: THP-1 cells treated with PMA at 5 ng/mL for 48 h to induce M0 differentiation (M0), the CM from induced M0 (M0-CM), sEVs extracted from M0 CM (M0-Exo), M0 treated with 30 mM glucose (high-glucose, HG) to induce M1 polarization (HG+M0), sEVs extracted from M0 treated with 30 mM glucose ((HG+M0)-Exo). The protein levels of sEVs markers, CD9, CD63, and CD81, were examined using Immunoblotting. **(C)** HUVECs were cultured with medium containing PKH67-labeled sEVs, non-labeled sEVs, or negative control samples and examined for the uptake of sEVs. Then, HUVECs were cultured with control culture medium (Con), medium containing sEVs extracted from PMA-treated THP-1 cells (M0-sEVs), medium containing sEVs extracted from M0 treated with 30 mM glucose ((HG+M0)-sEVs), or medium from M0 treated with 30 mM glucose and sEVs generation inhibitor GW4869 ((HG+M0)-sEVs-/-), and examined for HUVECs viability by CCK-8 assay **(D)**; tube formation capacity of HUVECs by tube formation assay **(E)**; HUVECs cell migration by Wound healing assay **(F)**; the protein levels of Ang1, Flk1, Vash1, TSP1I, ICAM-1, and VCAM-1 by Immunoblotting assay **(G)**. n=3, ** p<0.01 vs.M0 or control group. ## p<0.01 vs. (M0)-sEVs group. && p<0.01 vs. (HG+M0)-sEVs group.

Firstly, sEVs were extracted from M0 or HG-induced M1 macrophages to investigate whether sEVs mediate macrophage M1 polarization-induced HUVEC dysfunction. A transmission electron microscope (TEM) was used to observe extracted sEVs (**Figure 2A**). To confirm the EVs markers, protein samples were extracted from original M0 macrophages, M0 macrophages CM, sEVs extracted from M0 macrophages CM, M1 polarized macrophages CM, sEVs extracted from M1 polarized macrophages CM. Immunoblotting was used to examine the protein contents of sEVs markers, CD9, CD63, and CD81. The contaminant marker, Calnexin expression was also determined. As showed in **Figure 2B**, the isolated sEVs were negative for Calnexin. The levels of CD9, CD63, and CD81 were considerably greater in sEVs derived from M0 macrophages, M1 polarized macrophages, and sEVs extracted from M1 polarized macrophages, with the greatest levels seen in sEVs extracted from M1 polarized macrophages. sEVs secretion increases in M1-polarized macrophages.

HUVECs were cultured with a media supplemented with PKH67-labeled sEVs, non-labeled sEVs, or negative control (PKH67 dye only) specimens to investigate the sEVs uptake by HUVECs. **Figure 2C** showed that only when cultured with a medium containing PKH67-labeled sEVs, the PKH67 signal was observed, suggesting sEVs uptake by HUVECs. HUVECs were subsequently cultured with control culture medium (Con), medium containing sEVs extracted from M0 macrophage (M0- sEVs), medium containing sEVs extracted from M1 polarized macrophage ((HG+M0)- sEVs), or medium from M1 polarized macrophage but inhibited sEVs generation by GW4869 ((HG+M0)-sEVs-/-). **Figures 2D-F** shows that in M0-sEVs and (HG+M0)-sEVs-/- groups, cell viability, tube formation ability, and migratory capacity of HUVECs were significantly promoted, whereas (HG+M0)-sEVs significantly inhibited HUVEC viability, tube formation, and migration. Therefore, sEVs mediated M1 polarized macrophage-induced HUVEC dysfunction. Consistently, Ang1, Flk1, ICAM-1, and VCAM-1 protein contents dramatically increased, while Vash1 and TSP1I protein contents decreased within M0-sEVs and (HG+M0)-sEVs-/- groups; in (HG+M0)-sEVs group, Ang1, Flk1, ICAM-1, and VCAM-1 showed to be dramatically decreased, whereas Vash1 and TSP1I protein contents were increased (**Figure 2G**). These results indicate that sEVs from HG-stimulated macrophages could be transported to HUVECs and impair HUVEC functions.

### 3.3 M1 macrophage-derived sEVs miR-503 induces HUVEC dysfunctions

Given the critical role of miRNAs in DFU, the online dataset GSE86298 was analyzed to identify macrophage-associated miRNAs that potentially act on HUVECs. **Figures S1A, B** shows that in HG-induced M1 polarized macrophages, pri-miR-185, pri-miR-15b, pri-miR-95, pri-miR107, and pri-miR-503 were significantly differentially expressed (logFc > 0.1 or < -0.1, P < 0.05); among them, pri-miR-185, pri-miR-93, and pri-miR-15b were down-regulated, while pri-miR-503 and pri-miR-107 were up-regulated. According to another dataset, GSE74296, the mature miR-503, and miR-107 were up-regulated in HG-induced vascular endothelial cells (**Figures S1C, D**).

**Figure 3:**
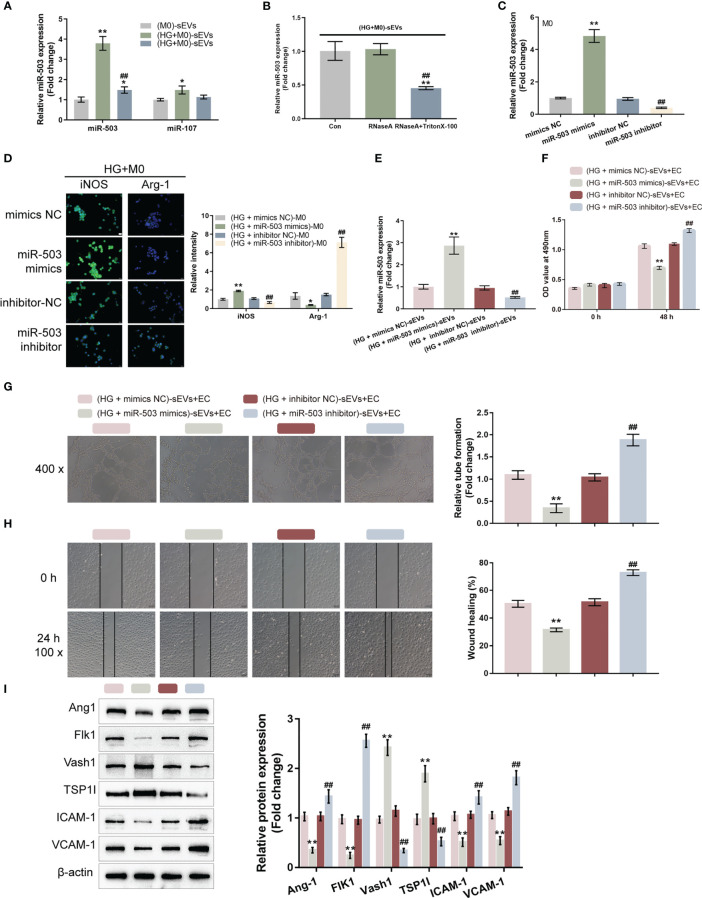
M1 macrophage-derived sEVs miR-503 induces HUVEC dysfunctions **(A)** The expression levels of miR-503 and miR-107 were examined in a medium containing sEVs extracted from PMA-treated THP-1 cells (M0-sEVs), medium containing sEVs extracted from M0 treated with 30 mM glucose ((HG+M0)-sEVs), or medium from M0 treated with 30 mM glucose and sEVs generation inhibitor GW4869 ((HG+M0)-sEVs-/-) using qRT-PCR. **(B)** sEVs extracted from M0 treated with 30 mM glucose ((HG+M0)-sEVs) were non-treated, treated with RNaseA, or treated with RNaseA plus TritonX-100 and examined for the expression level of miR-503 by qRT-PCR. **(C)** miR-503 overexpression or inhibition was achieved in macrophages by transfecting miR-503 mimics or inhibitor and confirmed using qRT-PCR. **(D)** M0 macrophages were transfected with miR-503 mimics or inhibitor, treated with HG, and examined for iNOS and Arg-1 levels by IF staining. **(E)** Then, M0 macrophages were transfected with miR-503 mimics or inhibitor, treated with HG, and extracted for sEVs. miR-503 expression in sEVs was determined by qRT-PCR. **(F-I)** HUVECs were cultured with sEVs from different groups and examined for cell viability by CCK-8 assay **(F)**; tube formation capacity **(G)**; cell migration by Wound healing **(H)**; the protein levels of Ang1, Flk1, Vash1, TSP1I, ICAM-1, and VCAM-1 by Immunoblotting assay **(I)**. n=3, ** p<0.01 vs.M0 (M0)-sEVs group or (HG+mimics NC)-sEVs group. ## p<0.01 vs. (HG+inhibitor NC)-sEVs group. * p<0.05.

Next, miR-503 and miR-107 expression within sEVs extracted from M0 macrophages were examined, sEVs extracted from M1 polarized macrophages ((HG+M0- sEVs), or sEVs generation inhibited M1 polarized macrophages medium ((HG+M0)-sEVs-/-). **Figure 3A** shows that miR-503 expression was dramatically elevated within (HG+M0)-sEVs and (HG+M0)-sEVs-/- groups, especially within (HG+M0)- sEVs. sEVs extracted from M1 polarized macrophages were non-treated, treated with RNaseA, or treated with RNaseA plus TritonX-100 and examined for miR-503 expression; as illustrated by **Figure 3B**, miR-503 expression was significantly decreased within the RNaseA plus TritonX-100 group, suggesting that extracellular miRNAs are mainly membrane-wrapped, as opposed to being released directly.

After confirming miR-503 upregulation in sEVs extracted from M1 polarized macrophages, miR-503 mimics/inhibitors were transfected to achieve miR-503 overexpression/inhibition in macrophages, as confirmed by qRT-PCR (**Figure 3C**). M0 macrophages were transfected with miR-503 mimics/inhibitor, treated with HG, and determined macrophage polarization by detecting iNOS and Arg-1 levels by IF staining. **Figure 3D** shows that, under HG stimulation, miR-503 overexpression significantly increased iNOS levels and decreased Arg-1 levels, whereas miR-503 inhibition decreased iNOS levels and increased Arg-1 levels. In addition, under normal glucose conditions, miR-503 overexpression could also promote M1 polarization (**Figure S2**). Therefore, miR-503 overexpression promoted macrophage M1 polarization.

M0 macrophages were subsequently transfected with miR-503 mimics or miR-503 inhibitors, treated with HG, and extracted for sEVs. miR-503 levels in sEVs were determined. As illustrated by **Figure 3E**, the miR-503 level was increased in sEVs from miR-503-overexpressed macrophages and was reduced in sEVs from miR-503-inhibited macrophages. HUVECs were subsequently cultured with sEVs from distinct groups and the HUVECs cell functions were determined. When cultured with sEVs from miR-503-overexpressed macrophages, HUVECs cell viability (**Figure 3F**), tube formation (**Figure 3G**), and migratory ability (**Figure 3H**) were significantly inhibited. When cultured with sEVs from miR-503-inhibited macrophages, HUVECs cell viability (**Figure 3F**), tube formation (**Figure 3G**), and migratory ability (**Figure 3H**) were significantly promoted. Thus, sEVs miR-503 from M1 polarized macrophages impairs HUVEC functions. Consistently, when cultured with sEVs from miR-503-overexpressed macrophages, the protein levels of Ang1, Flk1, ICAM-1, and VCAM-1 were decreased, but Vash1 and TSP1I were increased in HUVECs; when cultured with sEVs from miR-503-inhibited macrophages, Ang1, Flk1, ICAM-1, and VCAM-1 protein contents were increased, but Vash1 and TSP1I were decreased in HUVECs (**Figure 3I**). These results confirm that miR-503 was upregulated in sEVs from HG-stimulated macrophage associated with the HUVECs dysfunction.

### 3.4 Packaging of miR-503 into macrophage-derived sEVs is mediated by ACO1 protein

**Figure 4:**
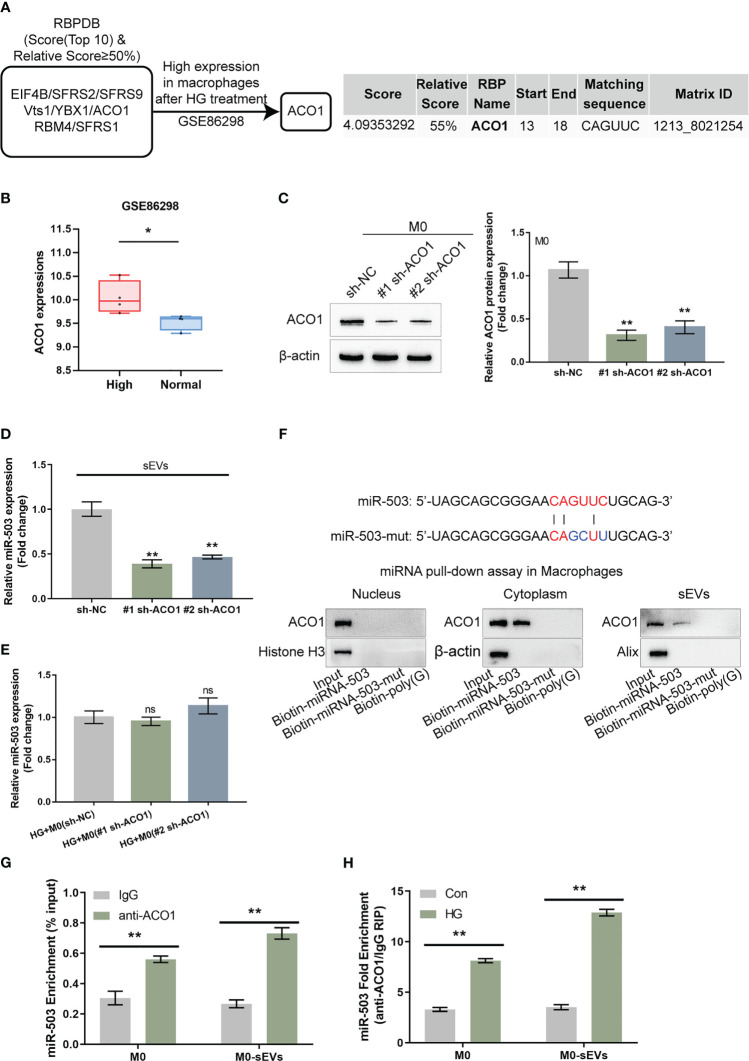
ACO1 protein mediates miR-503 packaging into macrophage-derived sEVs **(A)** The specific interaction between the miR-503 sequence and motifs of RNA-binding proteins (RBPs) was analyzed using the database of RBP specificities (RBPDB, http://rbpdb.ccbr.utoronto.ca/; threshold 0.7). Among 8 candidates, ACO1 was overexpressed in macrophages after HG treatment according to GSE86298. **(B)** ACO1 expression in HG-treated or non-treated macrophages according to GSE86298. **(C)** ACO1 knockdown was achieved in M0 macrophages by transfecting short hairpin RNA targeting ACO1 vector (#1 sh-ACO1 and #2 sh-ACO1) and confirmed by qRT-PCR. **(D)** sEVs were extracted from M0 macrophages transfected with sh-NC, #1 sh-ACO1, or #2 sh-ACO1 under HG or normal glucose condition for 72 h and examined for the expression of miR-503 by qRT-PCR. **(E)** M0 macrophages were treated with HG, transfected with sh-NC, #1 sh-ACO1, or #2 sh-ACO1, and examined for the cellular expression of miR-503 by qRT-PCR. **(F)** ACO1 levels were determined by Immunoblotting in samples derived by miRNA pulldowns using biotinylated miR-503 or mutated miR-503 in nuclear, cytoplasmic, or sEVs lysates. the biotinylated poly(G) was used as a negative control. **(G)** RIP assay of anti-IgG (negative control) or anti-ACO1 antibody was performed on cell or sEVs lysates from M0 macrophages. The level of miR-503 in the immunoprecipitated samples was measured by qRT-PCR and reported as a percentage relative to the input sample. **(H)** RIP assay of anti-IgG or anti-ACO1 antibody was performed on cell or sEVs lysates from M0 macrophages under normal glucose or HG condition. The level of miR-503 in the immunoprecipitated samples was measured by qRT-PCR and reported as a fold change of anti-ACO1/anti-IgG. N=3, ** p<0.01 vs. sh-NC group, IgG group or control group. * p<0.05; ns, no significance.

Given the theory that miRNAs could be packaged into sEVs in a motif of RNA-binding proteins (RBPs)-dependent manner, the possible crosstalk between the miR-503 sequence and motifs of RBPs was subsequently analyzed based on the RBP specificities database (RBPDB, http://rbpdb.ccbr.utoronto.ca/; threshold 0.7) (32). As demonstrated by the results, EIF4B, SFRS2, SFRS9, VTZ1, YBX1, ACO1, RBM4, and SFRS1 had specific binding sites of miR-503 with a relative score of ≥ 50% (**Figure 4A**). Moreover, according to GSE86298, ACO1 expression was significantly up-regulated in HG-induced macrophages (**Figures 4A, B**). Thus, ACO1 supposedly mediates miR-503 packaging into macrophage-derived sEVs.

Short hairpin RNA targeting ACO1 (#1 sh-ACO1 and #2 sh-ACO1) were subsequently transfected to achieve ACO1 knockdown within M0 macrophages, as confirmed by qRT-PCR (**Figure 4C**). EVs were extracted from M0 macrophages transfected with sh-NC, #1 sh-ACO1, or #2 sh-ACO1 and determined for the mRNA expression of ACO1; **Figure 4D** showed that sEVs miR-503 levels downregulated in #1 sh-ACO1 or #2 sh-ACO1 transfection of M0 macrophages both under HG or normal glucose. sEVs miR-503 from HG-stimulated macrophages was higher than the normal glucose one. Moreover, under HG condition, M0 macrophages transfected with sh-NC, #1 sh-ACO1, or #2 sh-ACO1, and determined for miR-503 expression within macrophages; as shown by **Figure 4E**, miR-503 levels in macrophages remained almost unaltered by ACO1 knockdown. These findings suggest that ACO1 might specifically affect miR-503 packaging into macrophage-derived sEVs.

A miRNA pulldown assay was then performed to investigate ACO1 interaction with miR-503 in the nucleus, cytoplasm, and sEVs. **Figure 4F** shows the cytoplasmic and sEVs interaction between ACO1 and miR-503; however, this interaction was not observed in the nucleus. When the mutation removed the CAGUUC sequence of miR-503, ACO1-binding capacity was eliminated (**Figure 4F**). Secondly, RNA immunoprecipitation (RIP) assays were performed in sEVs and cell lysates of M0 macrophage. miR-503 was found to be enriched within the ACO1 antibody group than the IgG (**Figure 4G**). Importantly, in RIP assays in the sEVs and cell lysates of HG-treated or non-treated M0 macrophages with anti-IgG, miR-503 was enriched in the HG-treated group compared to the non-treated group (**Figure 4H**), indicating that the interaction between ACO1 and miR-503 was increased in HG-induced M1 polarized macrophages.

### 3.5 sEVs miR-503 targets endothelial IGF1R and inhibits IGF1R expression

**Figure 5:**
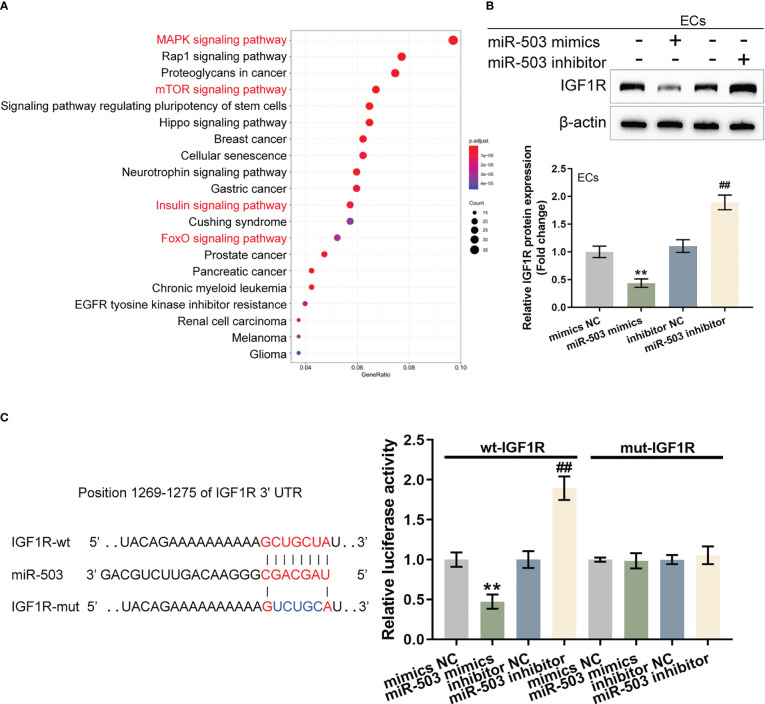
sEVs miR-503 targets endothelial IGF1R and inhibits IGF1R expression **(A)** Potential targets of miR-503 were predicted using miRDIP (https://ophid.utoronto.ca/mirDIP/) and predicted targets with high scores were applied for the Kyoto Encyclopedia of Genes and Genomes **(KEGG)** signaling pathway enrichment annotation. **(B)** HUVECs were transfected with miR-503 mimics or miR-503 inhibitors and examined for the protein levels of IGF1R were examined using Immunoblotting. **(C)** Wild- and mutant-type IGF1R 3’UTR luciferase reporter vectors were constructed and co-transduced into tool cells (293T) with miR-503 mimics or inhibitor. Luciferase activity was determined. N=3, ** p<0.01 vs. mimics NC group. ## p<0.01 vs. inhibitor NC group.

Given the theory that sEVs miR-503 could affect HUVEC functions, miRDIP (https://ophid.utoronto.ca/mirDIP/, last access date 3-24, 2021) was used to predict the putative targets of miR-503 and applied these predicted targets (**Table S3**) with a high score for the Kyoto Encyclopedia of Genes and Genomes (KEGG) signaling pathway enrichment annotation. **Figure 5A** illustrates the enrichment of its target genes in the MAPK pathway, insulin pathway, FOXO pathway, mTOR pathway, HIPPO pathway, and related totipotent stem cell maintenance. These key pathways are all related to endothelial cell proliferation, senescence, adhesion and migration (33–35).

miR-503 regulation of IGF1R was investigated as the key target gene IGF1R (located in the common upstream of the MAPK, insulin, FOXO, and mTOR pathways) is associated with cell proliferation and plays a protective role in activating vascular endothelial injury. HUVECs were transfected with miR-503 mimics/inhibitors and IGF1R protein contents were determined. **Figure 5B** shows that in HUVECs, miR-503 overexpression decreased IGF1R protein, whereas miR-503 inhibition increased IGF1R. Two different types of IGF1R luciferase reporter vectors, wild- and mutant-type, were subsequently constructed and these vectors were co-transduced into tool cells (293T) with miR-503 mimics or miR-503 inhibitors. The luciferase activity was subsequently analyzed. As shown by **Figure 5C**, in cells co-transfected with wild-type IGF1R 3’UTR reporter, miR-503 overexpression inhibited the luciferase activity, whereas miR-503 inhibition enhanced the luciferase activity. In cells co-transfected with mutant-type IGF1R 3’UTR reporter, miR-503 caused nearly no alterations within luciferase activity. These results indicated that miR-503 could target and modulate IGF1R expression *via* binding 3’UTR of IGF1R.

### 3.6 Dynamic effects of the miR-503/IGF1R axis upon HG-stimulated HUVEC function

**Figure 6:**
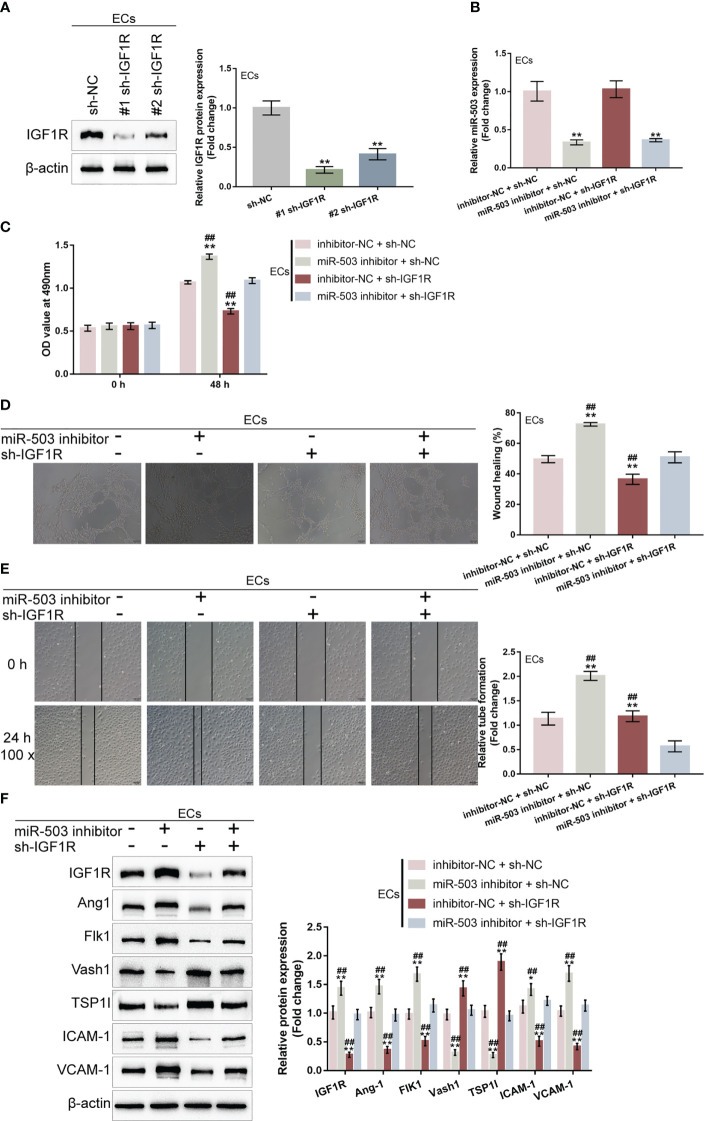
Dynamic effects of the miR-503/IGF1R axis on HG-stimulated HUVEC function **(A)** IGF1R knockdown was achieved in HUVECs by transfecting short hairpin RNA targeting IGF1R (#1 sh-IGF1R or #2 sh-IGF1R) and confirmed using Immunoblotting. Then, HUVECs were co-transduced with miR-503 inhibitor and sh-IGF1R and examined for miR-503 expression using qRT-PCR **(B)**; cell viability by CCK-8 assay **(C)**; tube formation capacity **(D)**; cell migration by Wound healing assay **(E)**; the protein levels of Ang1, Flk1, Vash1, TSP1I, ICAM-1, and VCAM-1 by Immunoblotting assay **(F)**. n=3, ** p<0.01 vs. sh-NC group. ## p<0.01 vs. miR-503 inhibitor+sh-IGF1R group.

Given that miR-503 targets IGF1R and inhibits IGF1R expression, next, the dynamic effects of the miR-503/IGF1R axis upon HG-stimulated HUVECs were investigated. Short hairpin RNA vector targeting IGF1R (#1 sh-IGF1R or #2 sh-IGF1R) was transfected to achieve IGF1R knockdown within HUVECs, as confirmed by Immunoblotting (**Figure 6A**). HUVECs were subsequently co-transduced with miR-503 inhibitor and sh-IGF1R and examined for miR-503 expression. **Figure 6B** showed that miR-503 inhibitor significantly downregulated miR-503 expression, whereas IGF1R knockdown caused no changes in miR-503 expression, indicating that IGF1R was downstream of miR-503. Consistently, miR-503 inhibition promoted HUVEC viability, tube formation capacity, and migratory ability, whereas IGF1R knockdown exerted opposite effects (**Figures 6C–E**); the promotive effects of miR-503 inhibition on HUVEC viability, tube formation capacity, and migratory ability were partially attenuated by IGF1R knockdown (**Figures 6C–E**). Consistently, miR-503 inhibition increased Ang1, Flk1, ICAM-1, and VCAM-1 protein contents but decreased Vash1 and TSP1I, whereas IGF1R knockdown decreased Ang1, Flk1, ICAM-1, and VCAM-1 but increased Vash1 and TSP1I (**Figure 6F**). Taken together, the miR-503/IGF1R axis could modulate HUVECs cell function.

### 3.7 *In vivo* effects of the miR-503/IGF1R axis upon diabetic mice wound healing

**Figure 7:**
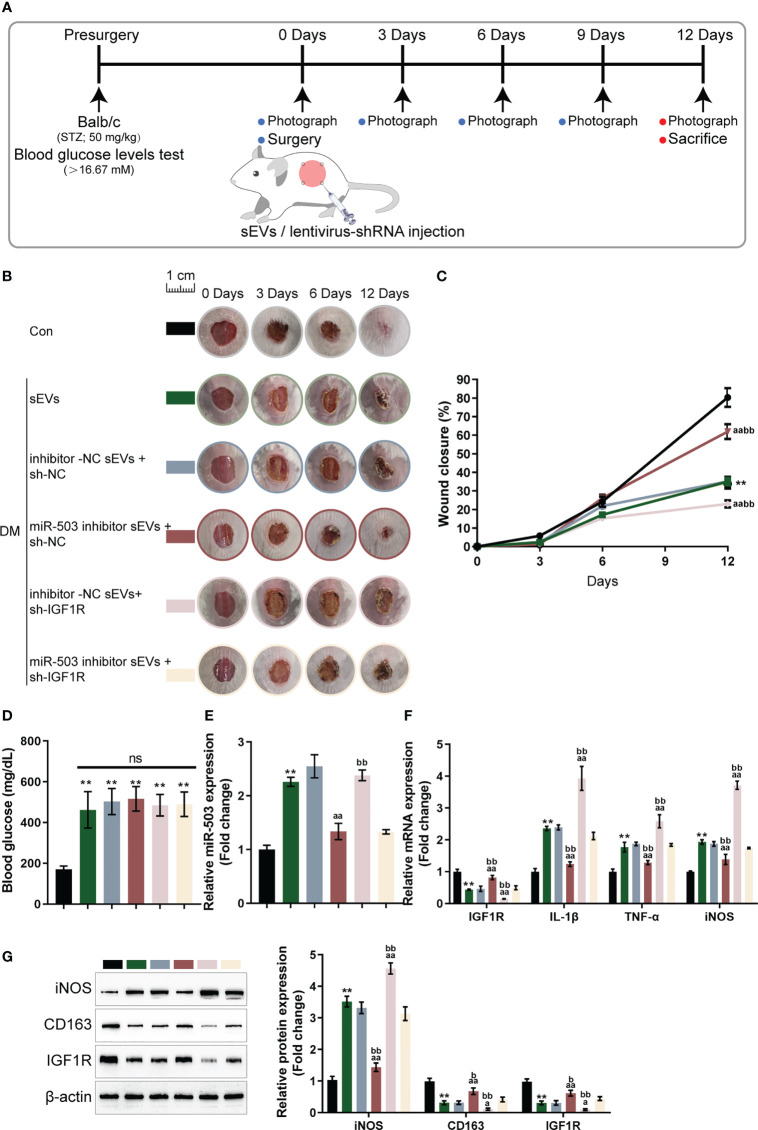
*In vivo* effects of the miR-503/IGF1R axis on diabetic mice wound healing **(A)** Mouse skin wound model was established in control or streptozotocin (STZ)-induced diabetic mice and divided into six groups: control, DM+sEVs, DM+inhibitor NC DM+sEVs + sh-NC, DM+miR-503 inhibitor sEVs + sh-NC, DM+inhibitor NC sEVs + sh-IGF1R, and DM+miR-503 inhibitor sEVs + sh-IGF1R. Mice in each group were treated accordingly. **(B)** The appearance of the wound on days 0, 3, 6, and 12 was shown. **(C)** Wound closure on day 0, 3, 6, and 12 was calculated. n=8. **(D)** The blood glucose were determined at day 12. n=8. **(E-G)** On day 12, mice were sacrificed, skin samples were collected, and the expression of miR-503, IGF1R, IL-1β, TNF-α, and iNOS were examined; the protein levels of iNOS, CD163, and IGF1R were examined using Immunoblotting **(G)**. n=3. ** p<0.01 vs. control group. aa p<0.01 vs. DM+inhibitor NC-sEVs+sh-NC group. bb p<0.01 vs. DM+miR-503 inhibitor -sEVs+sh-IGF1R group. * p<0.05. p<0.01 vs. control group. a p<0.05, aa p<0.01 vs. DM+inhibitor NC-sEVs+sh-NC group. b p<0.05, bb p<0.01 vs. DM+miR-503 inhibitor -sEVs+sh-IGF1R group.

Finally, the skin wound model was established in control or streptozotocin (STZ)-induced diabetic mice and divided into six groups: control, DM+ sEVs, DM+inhibitor NC sEVs + sh-NC, DM+ miR-503 inhibitor sEVs + sh-NC, DM+inhibitor NC sEVs + sh-IGF1R, and DM+miR-503 inhibitor sEVs + sh-IGF1R (**Figure 7A**). The sEVs from mouse macrophage RAW264.7 with mmu-miR-503 inhibitor transfection were identified (**Figures S3A, B**). The miR-503/IGF1R axis also existed in the mouse endothelial cell line C166 (ECs) (**Figures S3C, D**). Mice in each group were treated as per the aforementioned methods (**Figure 7A**). The appearances of the wound on days 0, 3, 6, and 12 were shown in **Figure 7B**. **Figure 7C** showed that the wound closure in the miR-503 inhibitor sEVs + sh-NC group was higher than any other groups, whereas that of the inhibitor NC sEVs + sh-IGF1R was the lowest. On day 12, the blood glucose was similar among DM mice with different sEVs or lentivirus injection (**Figure 7D**). Then, we sacrificed mice, collected skin samples, and examined the expression of miR-503, IGF1R, IL-1β, TNF-α, and iNOS. Firstly, in DM mice, miR-503 inhibitor sEVs+sh-NC and miR-503 inhibitor sEVs+sh-IGF1R reduced the miR-503 levels in the wound skin tissues compared to the inhibitor sEVs+sh-NC group. However, sh-IGF1R injection did not affect the miR-503 expression (**Figure 7E**). As shown by **Figure 7E**, IL-1β, TNF-α, and iNOS expression was dramatically downregulated in the miR-503 inhibitor sEVs + sh-NC group but up-regulated in the inhibitor-NC sEVs + sh-IGF1R group; in DM, inhibitor NC sEVs + sh-NC, and miR-503 inhibitor sEVs + sh-IGF1R groups, IL-1β, TNF-α, and iNOS expression was partially lower than the inhibitor NC sEVs + sh-IGF1R group. The expression trend of IGF1R was opposite to these factors (**Figure 7F**). Consistently, the protein levels of iNOS were dramatically upregulated within the miR-503 inhibitor sEVs + sh-NC group but downregulated within the inhibitor NC sEVs + sh-IGF1R group; IGF1R and CD163 protein levels were reduced in the miR-503 inhibitor sEVs + sh-NC group but increased in the inhibitor NC sEVs + sh-IGF1R group (**Figure 7G**). In the miR-503 inhibitor sEVs + sh-IGF1R group, the protein levels of iNOS, CD163, and IFG1R were almost reversed to the levels in the DM +sEVs and inhibitor NC sEVs + sh-NC groups (**Figure 7G**). These results indicate that sEVs miR-503 could potentially impair diabetic wound healing *via* inhibited IGF1R expression.

## 4 Discussion

In this study, macrophage M1 polarization was found to be dominant in DFU skin tissues; in HG-induced M1 polarized macrophages, iNOS was increased, whereas Arg-1 was decreased. Conditioned culture medium from HG-stimulated macrophages impaired HUVEC functions by inhibiting cell viability, tube formation, and cell migration. HG promotedsEVs secretion from M1 macrophages, and M1 macrophage-derived sEVs mediated M1 macrophage-caused HUVEC dysfunction. Under HG stimulation, sEVs miR-503 was up-regulated; inhibiting miR-503 in HG-stimulated macrophages attenuated M1 macrophage-caused HUVEC dysfunction. ACO1 interacted with miR-503 and mediated the miR-503 package into sEVs. Under HG stimulation, sEVs miR-503 taken in by HUVECs targeted IGF1R in HUVECs and inhibited IGF1R expression. In HUVECs, miR-503 inhibition improved HG-caused HUVEC dysfunction, whereas IGF1R knockdown aggravated HUVEC dysfunction; IGF1R knockdown partially attenuated miR-503 inhibition effects on HUVECs. In the skin wound model in control or STZ-induced diabetic mice, miR-503-inhibited sEVs improved, whereas IGF1R knockdown further hindered wound healing.

M1-like macrophage accumulation and excessive inflammation play a vital role in DFU pathogenesis (36). It has been shown that in diabetic wounds, the transition from M1-like to M2-like phenotypes is impeded, and the expression of M2-marker genes (such as Arg1) is drastically reduced (5). In this study, similar results were observed in DFU samples that iNOS, IL-6, and MCP-1 showed to be increased, but CD163, Arg-1, IL-4, and IL-10 showed to be decreased in DFU. As further evidence of the dominant M1 macrophage impairing wound healing, conditioned medium from HG-stimulated M1 macrophage significantly impaired HUVEC functions by inhibiting cell viability, tube formation capacity, and migratory cell ability. As aforementioned, HUVECs exert a crucial effect on the wound-healing process (15, 16); M1 macrophage-caused HUVEC dysfunction suggests that the dominant M1 polarization of macrophages contributes to impaired wound healing in DFU.

The sEVs carried non-coding RNAs, particularly miRNAs, exert an essential effect on inflammatory diseases. Reportedly, mesenchymal stromal cells (MSCs) could secret sEVs containing miRNAs, thus participating in promoted angiogenesis, mitigated oxidative stress and cell senscence, and playing a role in diabetic mouse wound healing model (37). Conversely, circulating sEVs from diabetic patients were abundant with miR-24-3p and miR-24-3p inhibition in circulating sEVs from diabetic patients enhanced HUVEC viability, tube formation capacity, and migratory ability (38). Macrophage-derived sEVs miRNAs have been reported to be involved in several biological processes, including tumor metastasis (39), myocardialfibrosis (40), vascular, bone or muscle trauma healing (41–43). Herein, HG stimulation on macrophages promoted the secretion of sEVs and the uptake of macrophage-derived sEVs by HUVECs, suggesting that macrophage-derived sEVs might mediate M1 macrophage-caused HUVEC dysfunctions. As surmised, incubation with M1 macrophage-derived sEVs caused similar impairment to HUVEC functions, whereas eliminating sEVs partially restored HUVEC functions.

Comprehensive bioinformatics analysis was subsequently performed to identify sEVs miRNAs that might mediate HUVEC dysfunctions, and miR-503 was found to be up-regulated in HG-stimulated macrophage. Previously, miR-503 has been reported to regulate endothelial changes caused by HG or hypoxia. Wen et al. (44) indicated that the upregulation of miR-503 suppressed hypoxia-induced EPC (endothelial progenitor cell) proliferative ability, migratory ability and capillary-like tube formation. Chen et al. (45) demonstrated HG-induced miR-503 upregulation in microvascular endothelial cells; miR-503 inhibition alleviates HG-induced microvascular endothelial cell inflammation and oxidative stress. Interestingly, as previously reported, endothelial-derived exosomal miR-503 could be transferred to tumor cells, thereby promoting the anti-tumor response of epirubicin (46). Herein, we found HG-induced up-regulation of sEVs miR-503. After inhibiting miR-503 in macrophages, macrophage M1 polarization was attenuated; moreover, sEVs derived from miR-503-inhibited M1 macrophages caused fewer HUVEC dysfunctions. Thus, increased sEVs miR-503 is supposed to be the reason for HUVEC dysfunctions under HG conditions.

To explain sEVs miRNA output thus far, some studies have observed motif-based RBP identification (47). For example, the specific short motifs in miRNAs (EXOmotifs) and sumoylated heterogeneous nuclear ribonucleoprotein A2B1 (hnRNPA2B1) is one of the controllers regulating miRNAs loading into sEVs. hnRNPA2B1 binds a specific subset of miRNAs through their EXOmotifs, with sumoylation tuning binding rate (48). The appearance of Synaptotagmin-binding cytoplasmic RNA-interaction protein (SYNCRIP, also known as hnRNP-q or NSAP1), another member of the hnRNP protein family, in hepatocyte sEVs is related to the control of specific miRNAs (49). In this study, through comparing RBPDB analyzed motifs of RBPs specific for miR-503 and HG-induced factors in macrophages, ACO1 was supposed to mediate miR-503 packaging into macrophage-derived sEVs. After knocking down ACO1 in macrophages, sEVs miR-503 but not macrophage miR-503 was down-regulated. Through direct interaction with miR-503, ACO1 mediates the package of miR-503 into sEVs.

miRNAs exert their biological functions through targeting different downstream targets. Regarding miR-503, previous studies have recognized Apelin-12 and Apelin as miR-503 downstream targets in HG-induced microvascular endothelial cells (45) or hypoxia-stimulated endothelial progenitor cells (44). Here, miRDIP-predicted miR-503 targets were applied for KEGG signaling pathway enrichment annotation and IGF1R attracted our attention because of its role in cell proliferation and vascular endothelial cell injury. The activation of the IGF-1/IGF1R signaling has been reported to mediate the promotive roles of Ginsenoside F1 in angiogenesis (50). Although IGF1R reduces the bioavailability of NO and promotes the capacity of *in situ* endothelium regeneration; regulation of IGF-1R in the endothelium might contribute to the effective treatment of diseases associated with vascular growth and repair (51). Interestingly, miR-503 expression within HUVECs is elevated in high-glucose conditions; miR-503 inhibits the expression level of IGF1R to reduce HUVEC migratory and proliferative abilities while promoting cell apoptosis (52). Similarly, in the present study, miR-503 targeted IGF1R and inhibited IGF1R expression. Under HG conditions, miR-503 inhibition attenuated HG-induced HUVEC dysfunctions, whereas IGF1R knockdown aggravated HG-induced HUVEC dysfunctions; IGF1R knockdown significantly attenuated the effects of miR-503 inhibition.

In a skin wound model with diabetic mice, sEVs from miR-503-inhibited macrophages promoted wound healing, whereas lentivirus expressing sh-IGF1R actually inhibited wound healing, corroborating our *in vitro* findings. These *in vivo* findings indicated that sEVs miR-503 targets IGF1R in ECs to modulate diabetic wound healing. In conclusion, M1 macrophage-derived sEVs miR-503 targets IGF1R in HUVECs, inhibiting IGF1R expression, leading to HUVEC dysfunctions, and hindering diabetic wound healing. The package of miR-503 into M1 macrophage-derived sEVs might be mediated by ACO1.

However, there are still limitations in the present study. There currently is no consensus on the best method to isolate sEVs (53). The standard isolation methods include ultracentrifugation, ultrafiltration, size-exclusion chromatography, precipitation, immunoaffinity, and microfluidic technologies. Different isolation methods yield variations in the concentration, purity and size of sEVs (54). Whether the different isolation methods will affect the sEVs function need to be further investigated.

## Data availability statement

The original contributions presented in the study are included in the article/**Supplementary Material**. Further inquiries can be directed to the corresponding author.

## Author contributions

Conceptualization: YL. Methodology: YH, JW, FH, LT, JM, and YL. Investigation: YH, JW, FH, LT, JM, and YL. Visualization: YH, JW, FH, LT, JM, and YL. Supervision: YL. Writing – original draft: YH, YL. Writing – review & editing: YH, JW, YL. All authors contributed to the article and approved the submitted version.

## Supplementary material

Supplementary Figure 1 Bioinformatic selection of miRNAs related to DFU. **(A, B)** online dataset GSE86298 was analyzed to identify miRNAs that might act on HUVECs. pri-miR-185, pri-miR-15b, pri-miR-95, pri-miR-107, and pri-miR-503 were significantly differentially expressed (logFc > 0.1 or < -0.1, P < 0.05). **(C, D)** In GSE74296, the mature miR-503 and miR-107 were up-regulated in HG-induced vascular endothelial cells.

Supplementary Figure 2 miR-503 promote M1 polarization under normal glucose condition. **(A)** M0 macrophages were transfected with miR-503 mimics or inhibitor, and examined for iNOS and Arg-1 levels by IF staining. **(B)** The levels of IL-6, MCP-1, IL-4 and IL-10 were determined by ELISA.

Supplementary Figure 3 Identification of sEVs from mouse macrophages. **(A)** the mouse macrophage cell line RAW264.7 was transfected with inhibitor-NC or mmu-miR-503 inhibitor and under HG condition. 72 h later, the sEVs were isolated and observed using TEM. **(B)** The protein levels of sEVs markers, CD9, CD63, and CD81, were examined using Immunoblotting. **(C,D)** Mouse ECs were transfected with mmu-miR-503 mimics or mmu-miR-503 inhibitors and examined for the protein levels of IGF1R were examined using Immunoblotting. **(C)** Wild- and mutant-type mmu-IGF1R 3’UTR luciferase reporter vectors were constructed and co-transduced into mouse ECs with mmu-miR-503 mimics or inhibitor. Luciferase activity was determined. ** p<0.01 vs. mimics NC group. ## p<0.01 vs. inhibitor NC group.

Supplementary Table 3 The miRDIP predicted target genes of miR-503
