# Supplementary material for: Diabetic macrophage small extracellular vesicles-associated miR-503/IGF1R axis regulates endothelial cell function and affects wound healing
Source: Front Immunol. 2023 May 23;14:1104890. doi: 10.3389/fimmu.2023.1104890 (PMC10243549; doi:10.3389/fimmu.2023.1104890)
Supplement: Supplementary file 1 [file Table_1.docx]

Table S1 the primer sequence for RT-PCR

| Gene Name | Forward 5’-3’ | Reverse 5’-3’ |
| --- | --- | --- |
| Hsa iNOS | TTCAGTATCACAACCTCAGCAAG | TGGACCTGCAAGTTAAAATCCC |
| Hsa Arg-1 | TGGACAGACTAGGAATTGGCA | CCAGTCCGTCAACATCAAAACT |
| Hsa β-actin | TTCCAGCCTTCCTTCCTGGG | TTGCGCTCAGGAGGAGCAAT |
| hsa miR-503-5p | RT：GTCGTATCCAGTGCGTGTCGTGGAGTCGGCAATTGCACTGGATACGACCTGCAG  F:TAGCAGCGGGAACAGTT | CAGTGCGTGTCGTGGA |
| hsa miR-107 | RT:  GTCGTATCCAGTGCGTGTCGTGGAGTCGGCAATTGCACTGGATACGACTGATAG  F:GAGCAGCATTGTACAGGG | CAGTGCGTGTCGTGGA |
| hsa U6 | CTCGCTTCGGCAGCACA | AACGCTTCACGAATTTGCGT |
| Mmu miR-503-5p | RT:  GTCGTATCCAGTGCGTGTCGTGGAGTCGGCAATTGCACTGGATACGACCTGCAG  F：TAGCAGCGGGAACAGTA | CAGTGCGTGTCGTGGA |
| mmu U6 | CTCGCTTCGGCAGCACA | AACGCTTCACGAATTTGCGT |
| mmu Arg1 | CTCCAAGCCAAAGTCCTTAGAG | AGGAGCTGTCATTAGGGACATC |
| mmu iNOS | GTTCTCAGCCCAACAATACAAGA | GTGGACGGGTCGATGTCAC |
| mmu IGF1R | CTTCTACAACTACGCACTGGTC | TCGGCGTTCTTCTCAATCCTG |
| mmu TNF-a | CCCTCACACTCAGATCATCTTCT | GCTACGACGTGGGCTACAG |
| mmu β-actin | GGCTGTATTCCCCTCCATCG | CCAGTTGGTAACAATGCCATGT |

Table S2 the sequence for mimics, inhibitor, shRNA and vector construction

| Gene Name | Forward 5’-3’ | Reverse 5’-3’ |
| --- | --- | --- |
| mimics NC | UUCUCCGAACGUGUCACGUTT | ACGUGACACGUUCGGAGAATT |
| Inhibitor NC | CAGUACUUUUGUGUAGUACAA |  |
| hsa miR-503-5p mimics | UAGCAGCGGGAACAGUUCUGCAG | GCAGAACUGUUCCCGCUGCUAUU |
| hsa miR-503-5p inhibitor | CUGCAGAACUGUUCCCGCUGCUA |  |
| hsa #1 sh-ACO1 | GATCCGGAATGTTTCGAGATTTCAATCTCGAGATTGAAATCTCGAAACATTCCTTTTTG | AATTCAAAAAGGAATGTTTCGAGATTTCAATCTCGAGATTGAAATCTCGAAACATTCCG |
| hsa #2 sh-ACO1 | GATCCGTATTTCTGAAAGATATCTGGCTCGAGCCAGATATCTTTCAGAAATACTTTTTG | AATTCAAAAAGTATTTCTGAAAGATATCTGGCTCGAGCCAGATATCTTTCAGAAATACG |
| Wt-IGF1R 3’UTR (human) | aattctaggcgatcgctcgagATTTTCTCTGTTCCTAGGACTTCTTCA | attttattgcggccagcggccgcAGAAGTATCCATTACCGGGAAAAG |
| Mut-IGF1R 3’UTR (human) | AgtctgcaTTTTTTTTGTTCTTGATCTTTGTGG | CAAAAAAAAtgcagacTTTTTTTTTTCTGTACAGACGTATAGATG |
| hsa #1 sh-IGF1R | GATCCGGAATTACTCCTTCTACGTCCCTCGAGGGACGTAGAAGGAGTAATTCCTTTTTG | AATTCAAAAAGGAATTACTCCTTCTACGTCCCTCGAGGGACGTAGAAGGAGTAATTCCG |
| hsa #2 sh-IGF1R | GATCCGATTCTAATGTATGAAATAAACTCGAGTTTATTTCATACATTAGAATCTTTTTG | AATTCAAAAAGATTCTAATGTATGAAATAAACTCGAGTTTATTTCATACATTAGAATCG |
| mmu #1 sh-IGF1R | GATCCGCAATCTGCTTATTAACATCCCTCGAGGGATGTTAATAAGCAGATTGCTTTTTG | AATTCAAAAAGCAATCTGCTTATTAACATCCCTCGAGGGATGTTAATAAGCAGATTGCG |
| mmu #2 sh-IGF1R | GATCCGCGTGAAAGAATCGAGTTTCTCTCGAGAGAAACTCGATTCTTTCACGCTTTTTG | AATTCAAAAAGCGTGAAAGAATCGAGTTTCTCTCGAGAGAAACTCGATTCTTTCACGCG |
| mmu miR-503-5p mimics | UAGCAGCGGGAACAGUACUGCAG | GCAGUACUGUUCCCGCUGCUAUU |
| mmu miR-503-5p inhibitor | CUGCAGUACUGUUCCCGCUGCUA |  |
| Wt-IGF1R 3’UTR (mouse) | aattctaggcgatcgctcgagATGGTCTGTTACTAGGACTTCTTCATGG | attttattgcggccagcggccgcCTAGAAGCATCAGTTGCCGGA |
| Mut-IGF1R 3’UTR (mouse) | GAAAAGAAAAgtctgcaTTTTTTTTTTGTTCTTTATCTTTGTGG | tgcagacTTTTCTTTTCCTGTACAGATGTATAGATGA |
