# Supplementary figures and images for: Diabetic macrophage small extracellular vesicles-associated miR-503/IGF1R axis regulates endothelial cell function and affects wound healing
Source: Front Immunol. 2023 May 23;14:1104890. doi: 10.3389/fimmu.2023.1104890 (PMC10243549; doi:10.3389/fimmu.2023.1104890)

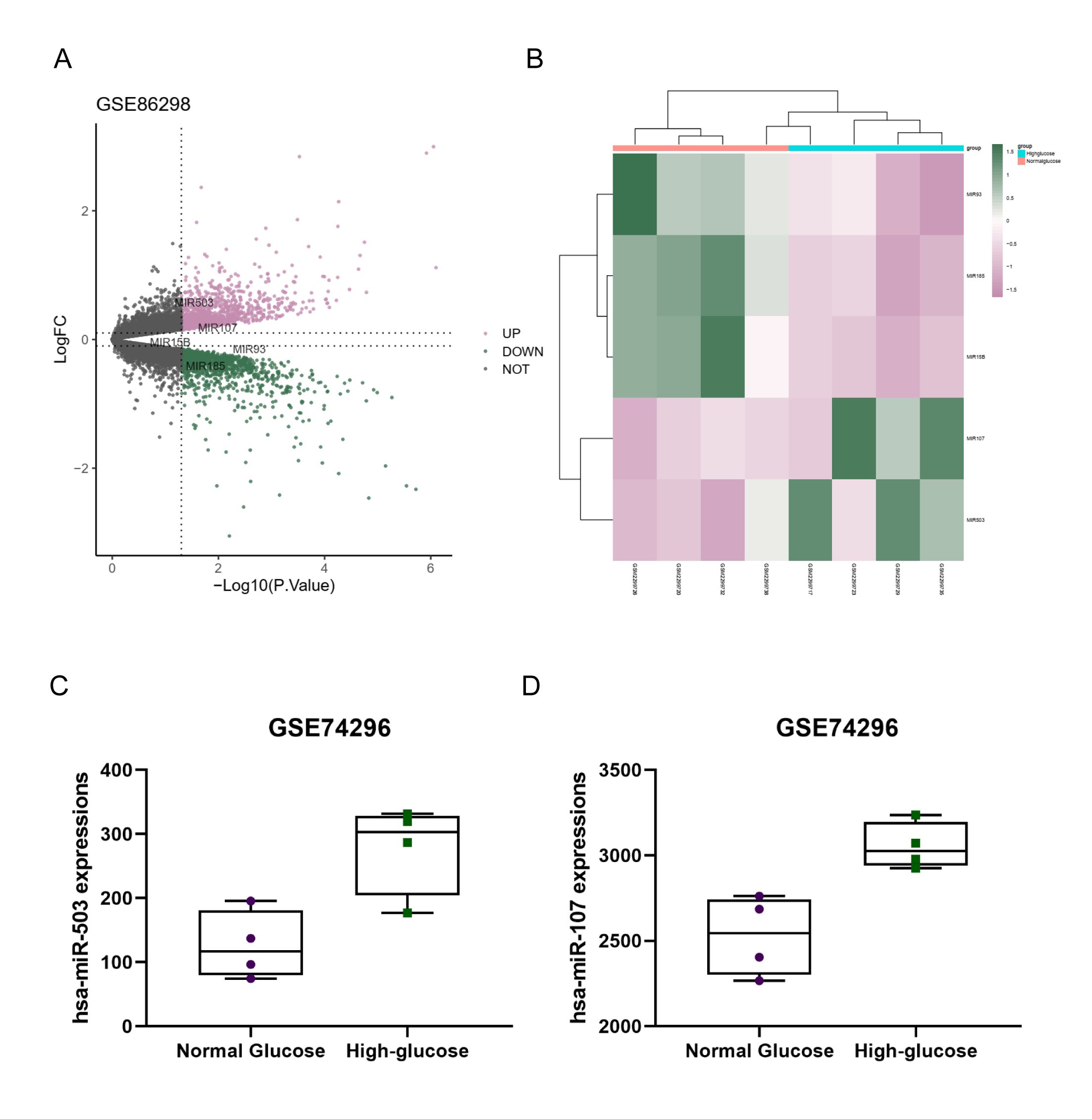

Supplement: Supplementary Figure 1 — Bioinformatic selection of miRNAs related to DFU. (A, B) online dataset GSE86298 was analyzed to identify miRNAs that might act on HUVECs. pri-miR-185, pri-miR-15b, pri-miR-95, pri-miR-107, and pri-miR-503 were significantly differentially expressed (logFc > 0.1 or < -0.1, P < 0.05). (C, D) In GSE74296, the mature miR-503 and miR-107 were up-regulated in HG-induced vascular endothelial cells. [file Image_1.tif]

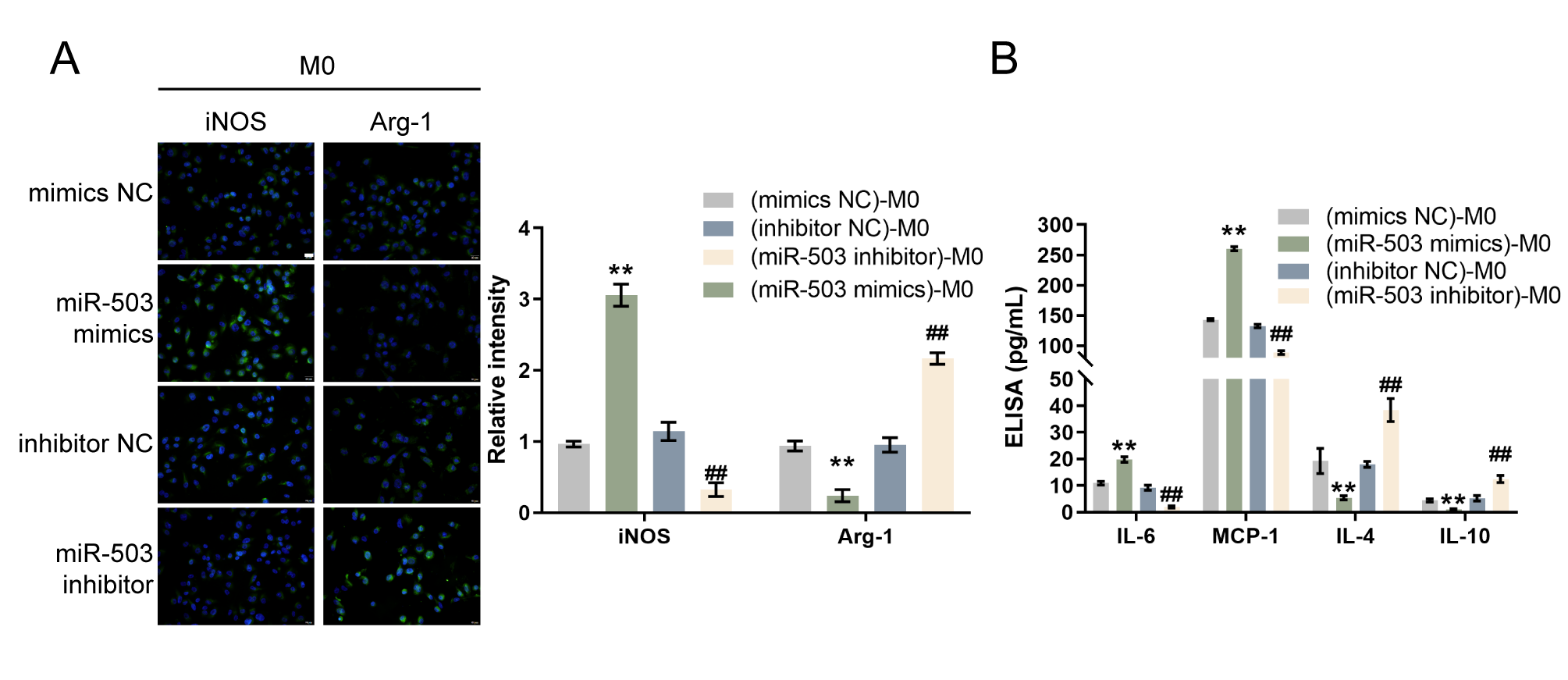

Supplement: Supplementary Figure 2 — miR-503 promote M1 polarization under normal glucose condition. (A) M0 macrophages were transfected with miR-503 mimics or inhibitor, and examined for iNOS and Arg-1 levels by IF staining. (B) The levels of IL-6, MCP-1, IL-4 and IL-10 were determined by ELISA. [file Image_2.tif]

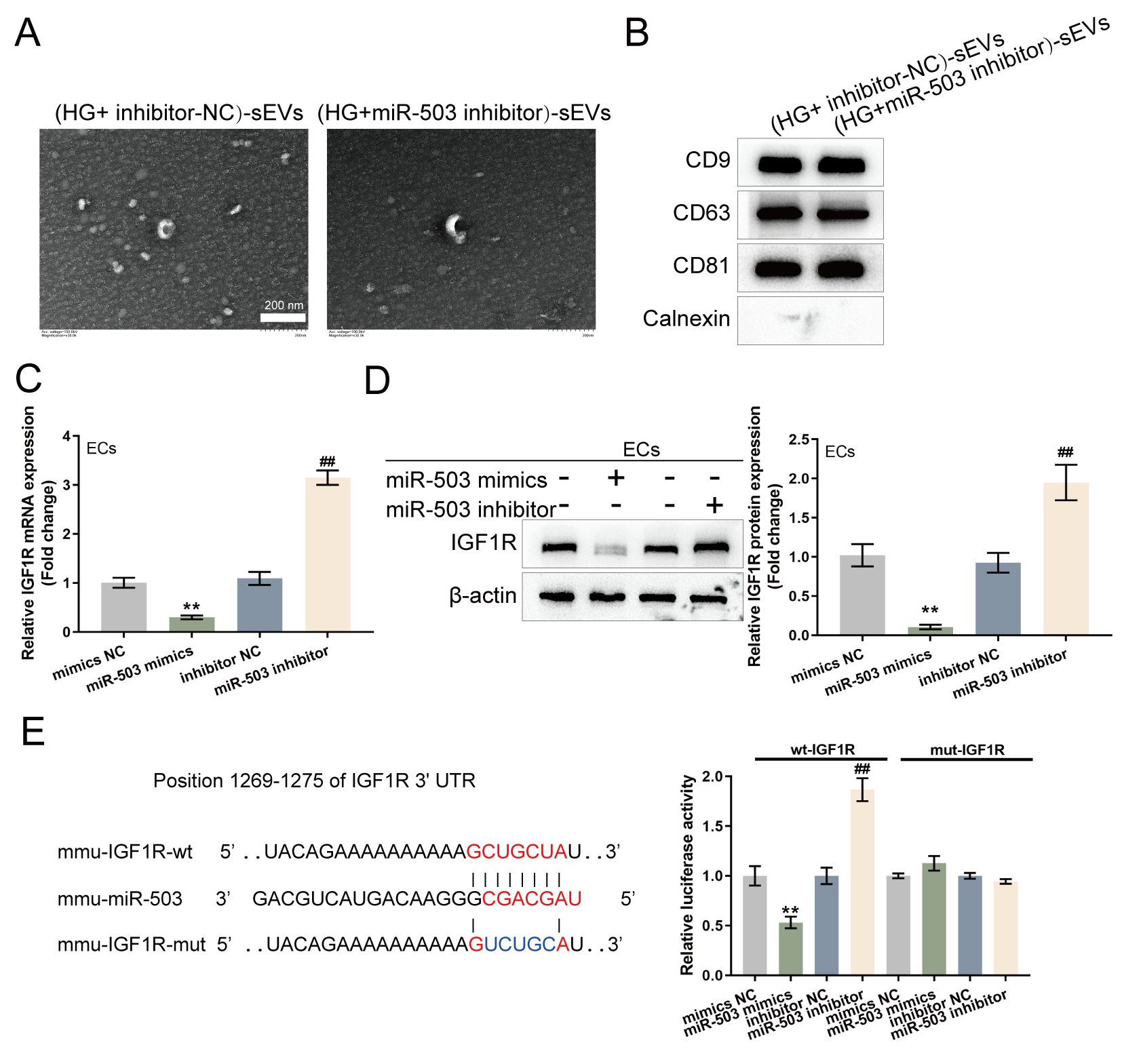

Supplement: Supplementary Figure 3 — Identification of sEVs from mouse macrophages. (A) the mouse macrophage cell line RAW264.7 was transfected with inhibitor-NC or mmu-miR-503 inhibitor and under HG condition. 72 h later, the sEVs were isolated and observed using TEM. (B) The protein levels of sEVs markers, CD9, CD63, and CD81, were examined using Immunoblotting. (C,D) Mouse ECs were transfected with mmu-miR-503 mimics or mmu-miR-503 inhibitors and examined for the protein levels of IGF1R were examined using Immunoblotting. (C) Wild- and mutant-type mmu-IGF1R 3’UTR luciferase reporter vectors were constructed and co-transduced into mouse ECs with mmu-miR-503 mimics or inhibitor. Luciferase activity was determined. ** p<0.01 vs. mimics NC group. ## p<0.01 vs. inhibitor NC group. [file Image_3.tif]
